# Impact of Body Mass Index on Functional Recovery After Total Hip Arthroplasty: A Prospective Study

**DOI:** 10.1016/j.artd.2026.101986

**Published:** 2026-03-07

**Authors:** Musashi Ima, Tamon Kabata, Daisuke Inoue, Yu Yanagi, Takahiro Iyobe, Naoya Fujimaru, Satoru Demura

**Affiliations:** Department of Orthopedic Surgery, Graduate School of Medical Science, Kanazawa University, Kanazawa, Japan

**Keywords:** Body mass index, Gait analysis, Obesity, Total hip arthroplasty

## Abstract

**Background:**

The effect of body mass index (BMI) on walking recovery after total hip arthroplasty (THA) has not yet been investigated using both clinical evaluation and biomechanics. The aim of this study was to examine the effects of BMI on functional recovery at 6 months and 1 year post-THA among normal-weight, overweight, and obese individuals based on preoperative status.

**Methods:**

This prospective case-control study involved 269 patients who underwent primary THA. Participants were categorized into normal-weight, overweight, and obese groups based on the World Health Organization criteria. Recovery outcomes were assessed across BMI groups using gait measurements and the Japanese Orthopaedic Association hip scores.

**Results:**

Patients with obesity exhibited slower recovery in walking speed and stride length at both 6 months and 1 year compared with normal-weight individuals and overweight patients. At 1 year postsurgery, the Japanese Orthopaedic Association hip scores showed no significant differences among the BMI groups, thereby indicating similar satisfaction levels despite initial functional recovery differences.

**Conclusions:**

Although patients with obesity faced early recovery challenges, particularly regarding gait, the level of satisfaction with THA outcomes was comparable across all BMI groups at 1 year. These findings highlight the need for personalized management and rehabilitation strategies for optimizing THA outcomes in patients with obesity.

## Introduction

Total hip arthroplasty (THA) is a highly effective treatment for restoring hip joint function in patients with hip osteoarthritis (OA) [1]. The number of THA procedures performed annually is increasing and is expected to rise further [2]. OA may result from congenital factors, such as developmental dysplasia of the hip and femoroacetabular impingement, and acquired factors, including heavy lifting, obesity, and sports activities [3]. Overweight and obesity are known risk factors for OA, and as their prevalence rises, so does the incidence of secondary OA of the hip [4,5]. THA in patients with obesity presents challenges, including increased risks of dislocation, deep infection, bleeding, deep vein thrombosis, and the need for revision surgery [[6], [7], [8], [9]]. Moreover, patients with obesity tend to have longer surgical periods and hospital stays [10]. From a biomechanical perspective, obesity is associated with reduced hip range of motion [11] and walking speed [[12], [13], [14], [15]].

The effect of obesity on the functional recovery of patients with OA is poorly understood. Some studies have shown that patients with obesity can achieve functional scores similar to those of patients with no obesity [16], whereas others have reported poorer outcomes [17]. Shibuya et al. reported that the maximal walking speed measured over a 10-m walk as fast as safely possible is a prognostic indicator of functional recovery after THA [18], Foucher established clinically meaningful benchmarks for gait improvement after THA [19], and Ohmori et al. reported that a comfortable walking speed of ≥1.34 m/s at 1 year post-THA indicates favorable long-term functional recovery [20]. Gait evaluation after THA has been widely used to assess postoperative functional recovery [[18], [19], [20]]. Previous studies have shown that gait speed and stride length gradually improve within the first postoperative year, reflecting objective recovery of mobility [15,18,19]. These gait parameters provide important biomechanical information that is not captured by patient-reported outcomes alone [11,19]. To date, no studies have investigated the effect of body mass index (BMI) on walking recovery after THA using both clinical evaluation and biomechanics. In this study, we aimed to explore the functional recovery differences at 6 months and 1 year post-THA among normal-weight, overweight, and obese groups based on preoperative status.

## Material and methods

### Patient selection

This prospective case-control study was approved by the Ethics Committee of Kanazawa University (approval number: 3669). All procedures were conducted in accordance with the ethical standards set forth in the 1964 Declaration of Helsinki and its later amendments or comparable ethical standards. Written informed consent was obtained from all patients before participation. Between December 11, 2015, and December 7, 2022, 320 cases underwent primary THA for OA at our institution. Patients with osteonecrosis of the femoral head, inflammatory hip disease, severe hip deformities such as Crowe types 3 and 4, and bilateral THAs were excluded, resulting in 269 hips included in the study.

All patient information was extracted from electronic medical records, and the patients were classified into 3 groups according to World Health Organization criteria. Preoperative radiographic evaluations were reviewed to assess adjacent joint conditions, including the ipsilateral knee, ankle, and lumbar spine. Patients with lumbar spine degenerative changes on imaging considered likely to affect gait function—such as marked disc space narrowing, prominent osteophyte formation, or segmental instability—were excluded, thereby minimizing the confounding effects of adjacent joint or spinal pathology on gait assessment.

Individuals with BMI < 25 kg/m^2^ (including underweight patients with BMI < 18.5 kg/m^2^) were classified as being normal weight, those with BMI 25-29.9 kg/m^2^ as being overweight, and individuals with BMI ≥ 30 kg/m^2^ as being obese.

### Implant positioning assessment

**Figure 1:**
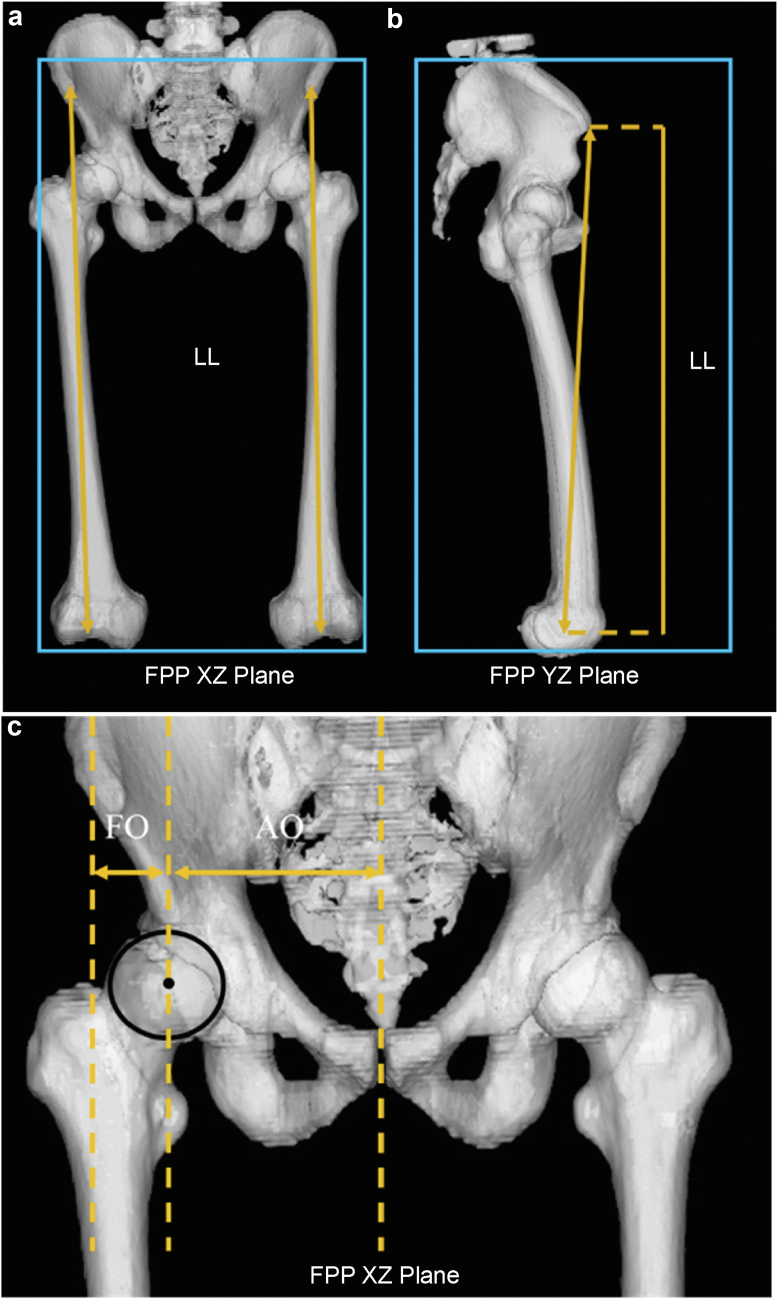
Three-dimensional computed tomography analysis. Leg length (LL), defined as the distance projected onto the frontal plane projection (FPP) from the anterior superior iliac spine to the center of the knee in the XZ plane (a) and YZ plane (b). Both the femoral offset (FO) and acetabular offset (AO) are defined as the lengths projected onto the FPP XZ plane in the neutral position (c). FO is the distance between the center of the femoral head, the center of the cup, and the axis of the femur. AO is the distance between the pubic symphysis and the center of the femoral head or cup. The global offset is the sum of FO and AO.

Preoperative and 1-week postoperative computed tomography (CT) scans were obtained for each patient. The CT images covered the area from the iliac crest to the femoral condyles, with slices cut at a thickness of 1 mm and a pitch of 2.5 mm, generating 160-250 slices per patient based on body size. All CT slices were imported into CT-based templating software (ZedHip, Lexi, Tokyo, Japan) in the Digital Imaging and Communications in Medicine format to create virtual 3-dimensional bone models for virtual surgery. This software enabled the accurate measurement of various parameters such as leg length and femoral and acetabular offsets [20,21] (Fig. 1).

### Surgical technique

All surgeries were performed by a single surgeon using an anterolateral approach. The cup was positioned to replicate the center of the contralateral femoral head and medialized until it contacted the teardrop. The femoral stem was inserted such that its offset increased in proportion to cup medialization, matching the global offset with the contralateral side.

### Patient-reported outcome measure assessment

The Japanese Orthopaedic Association (JOA) hip score was recorded preoperatively, at 6 months, and at 1 year postoperatively.

#### Gait assessment

The time required to walk 10 m was measured after a 2-m lead-in (acceleration) phase at a comfortable speed. The time taken to walk 10 m was recorded, and walking speed (m/s) was calculated. Additionally, the stride length (m) was calculated from the number of steps required to cover a 10-m distance [20].

#### Statistical analysis

All statistical analyses were performed using SPSS Statistics version 26 (IBM Corp., Armonk, NY, USA). Continuous variables, including age, BMI, operative time, blood loss, and CT parameters (leg length discrepancy, acetabular offset, and femoral offset), were compared among the 3 BMI groups using one-way analysis of variance. When the normality assumption was not met, the Kruskal–Wallis test was applied. Categorical variables such as sex and American Society of Anesthesiologists status were analyzed using the chi-square test.

One-way analysis of variance with repeated measures was used to compare walking speed, stride length, and the JOA hip score between the groups over time. When multiple comparisons were performed, Bonferroni correction was applied. Statistical significance was set at *P* < .05.

## Results

### Patient background

Patients with hip OA were classified into the BMI groups as shown in Table 1. No significant differences were found among the groups, except for BMI and surgical time.

**Table 1:** Patient background.

Variables	Normal-weight group	Overweight group	Obese group	*P* value
Age (y)	64.3 ± 8.8	66.1 ± 9.0	64.1 ± 8.7	.34
Sex (female/male)	162/15	61/7	24/1	.28
BMI	21.7 ± 2.0	27.1 ± 1.4	33.9 ± 2.7	<.05
Operation time (min)	154.5 ± 32.5	157.5 ± 44.6	186.3 ± 21.1	<.05
Postoperative day	17.3 ± 4.6	17.2 ± 3.5	20.5 ± 3.4	.06
ASA score	16/150/11	0/67/1	0/21/3	.11
Blood loss	219.1 ± 135.8	227.7 ± 144.2	262.5 ± 123.64	.34

ASA, American Society of Anesthesiologists; Operation time, time required for surgery; Postoperative day, period of hospitalization; Blood loss, intraoperative blood loss.

Table 2 presents the preoperative measurements of the leg length discrepancy and offset. No significant differences in leg length discrepancy, acetabular offset, or femoral offset were observed preoperatively or postoperatively.

**Table 2:** Computed tomography data analysis.

Variable	Group	Preoperative	Postoperative	*P* (pre vs post)	*P* (between groups, pre)	*P* (between groups, post)
Leg length discrepancy (mm)	Normal weight	−8.7 ± 11.0	1.7 ± 6.7	<.0001	.87	.42
	Overweight	−8.7 ± 10.4	0.5 ± 6.2	<.0001		
	Obese	−7.5 ± 9.6	1.0 ± 5.1	<.0001		
Acetabular offset (mm)	Normal weight	96.9 ± 9.0	88.5 ± 4.8	<.0001	.87	.42
	Overweight	96.3 ± 7.1	88.0 ± 4.1	<.0001		
	Obese	97.4 ± 6.3	88.3 ± 5.2	<.0001		
Femoral offset (mm)	Normal weight	27.6 ± 7.2	33.1 ± 6.2	<.0001	.45	.95
	Overweight	27.8 ± 7.1	32.8 ± 4.8	<.0001		
	Obese	29.6 ± 7.4	32.9 ± 7.5	<.0001		

### JOA hip score

**Figure 2:**
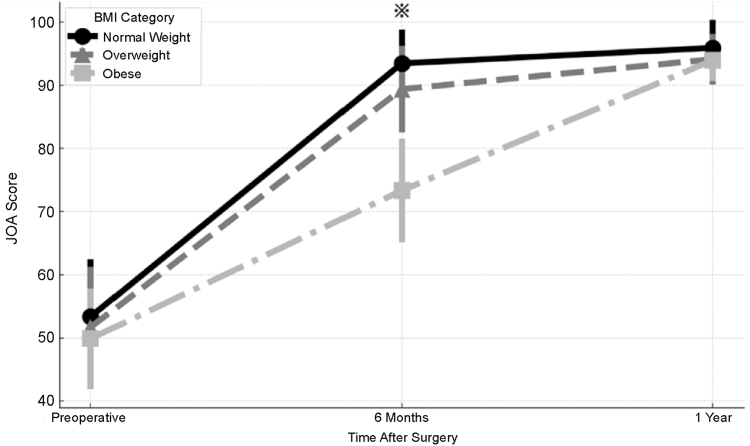
Japanese Orthopaedic Association (JOA) hip scores in the normal-weight, overweight, and obese groups at the preoperative and 6-month and 1-year postoperative time points.

Figure 2 shows the trajectory of the JOA hip score preoperatively, 6 months postoperatively, and 1 year postoperatively. Preoperatively, no significant between-group differences were observed among the 3 BMI groups (between-group *P* = .87). At 6 months postoperatively, the obese group showed significantly lower JOA scores than the normal-weight and overweight groups (both *P* < .05). However, at 1 year postoperatively, no significant differences were found among the 3 groups (*P* = .42), indicating that clinical recovery eventually equalized across the BMI groups.

No significant between-group differences were observed preoperatively (*P* = .87) or at 1 year postoperatively (*P* = .42).

However, at 6 months postoperatively, the obese group showed significantly lower JOA scores than both the normal weight and overweight groups (both *P* < .05).

Error bars represent standard deviations.

### Gait analysis

**Figure 3:**
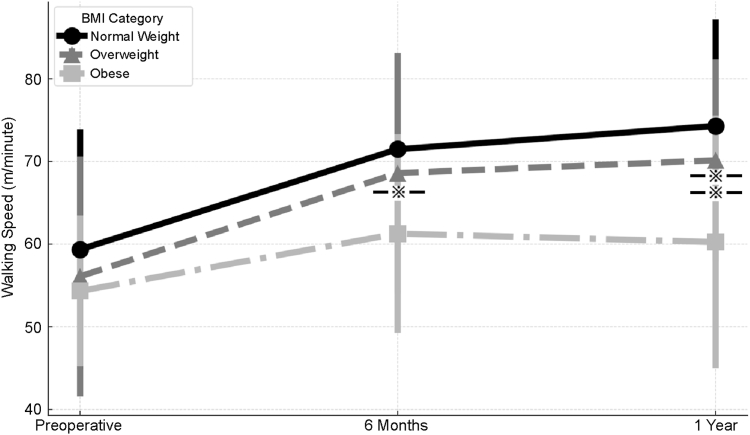
Walking speed in the normal-weight, overweight, and obese groups at the preoperative and 6-month and 1-year postoperative time points. No significant between-group differences were observed preoperatively. At 6 months postoperatively, the obese group showed significantly slower walking speed than the normal-weight group (*P* < .05). At 1 year postoperatively, the obese group showed significantly slower walking speed than both the normal-weight and overweight groups (*P* < .05). Error bars represent standard deviations. Asterisks indicate significant between-group differences.

**Figure 4:**
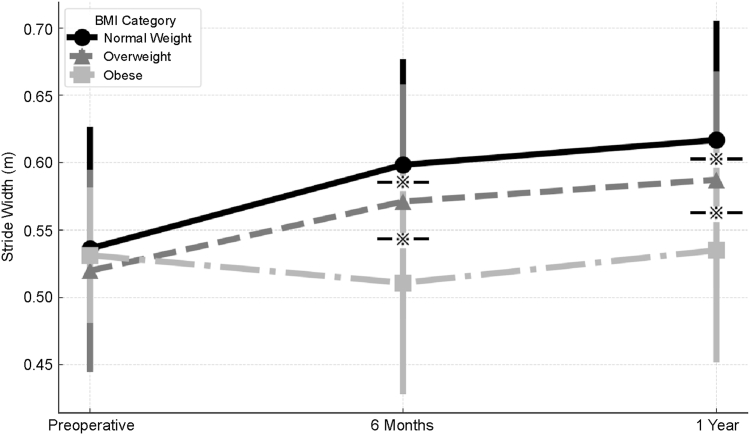
Stride length in the normal-weight, overweight, and obese groups at the preoperative and 6-month and 1-year postoperative time points. No significant between-group differences were observed preoperatively. At both 6 months and 1 year postoperatively, the obese group demonstrated significantly shorter stride length than the normal-weight and overweight groups (*P* < .05). Error bars represent standard deviations. Asterisks indicate significant between-group differences.

Figures 3 and 4 show the gait analyses preoperatively, 6 months postoperatively, and 1 year postoperatively. Preoperatively, no significant between-group differences were observed in either walking speed or stride length. Regarding walking speed, the obese group demonstrated significantly slower walking speed than the normal-weight group at 6 months postoperatively (*P* < .05). At 1 year postoperatively, the obese group continued to exhibit significantly slower walking speed than both the normal-weight and overweight groups (*P* < .05). Similarly, for stride length, the obese group showed significantly shorter stride length than the normal-weight and overweight groups at both 6 months and 1 year postoperatively (*P* < .05). Additionally, Δ analyses (defined as the change from the preoperative to the 1-year postoperative values) demonstrated that the improvement in gait parameters (walking speed and stride length) from preoperatively to 1 year postoperatively was significantly smaller in the obese group compared with the normal-weight and overweight groups (both *P* < .05).

## Discussion

This study demonstrated that although the obese group showed significantly slower gait recovery at 6 months and 1 year after THA, no significant differences in JOA hip scores were observed among the BMI groups at 1 year. These findings suggest that subjective clinical recovery—including pain and daily functional limitations—equalizes across BMI groups over time, whereas gait-specific functional deficits may persist, particularly in patients with obesity.

### Functional recovery and outcome evaluation in patients with obesity after THA

Although an increase in BMI is known to be associated with postoperative complications and delayed recovery of walking ability, our results showed that the JOA hip scores at 1 year postsurgery demonstrated no significant differences among the BMI groups.

This finding suggests that patients with obesity can ultimately achieve functional recovery comparable to that of individuals with normal body weight. This highlights that subjective clinical improvement tends to equalize despite early postoperative differences. Furthermore, THA provides sustained clinical benefit regardless of BMI by effectively reducing pain and improving joint mobility.

Previous studies have shown that obesity imposes increased physical load and reduced exercise tolerance, which may delay early rehabilitation and exacerbate postoperative difficulties [[22], [23], [24]]. These factors may partially explain the inferior gait performance observed in the obese group during the early postoperative period. In our cohort, the JOA pain subdomain did not differ significantly among the BMI groups, suggesting that postoperative pain alone does not account for the persistent gait deficits observed in the obese group. Although a separate visual analog scale was not used in this study, the JOA hip score includes a 40-point pain subdomain, and therefore, captures some degree of pain-related postoperative limitation. Future investigations should incorporate gait-related pain assessments to better clarify the contribution of postoperative pain to delayed gait recovery in patients with obesity.

The JOA score mainly reflects improvements in pain and daily living activities [25], which tend to recover earlier than objective gait parameters. These findings underscore the distinction between subjective clinical improvement and objective functional recovery and highlight the importance of evaluating both when assessing postoperative outcomes in patients with obesity undergoing THA.

### Challenges and strategies in rehabilitation for patients with obesity after THA

In the present study, the gait analysis showed that the obese group had inferior walking ability compared with the other BMI groups at both 6 months and 1 year postoperatively, indicating that obesity significantly impedes recovery of walking function [12,18]. Obesity increases mechanical loading on the hip joint, reduces hip abductor strength, and impairs balance control, all of which can contribute to slower improvements in walking speed and stride length. These gait deficits can substantially affect activity levels and daily life independence, highlighting the need for rehabilitation programs tailored specifically to the limitations associated with obesity.

In the short-term recovery phase, focused strategies including pain management, joint protection, and muscle strength maintenance are essential for mitigating exercise intolerance and early postoperative discomfort [26,27]. Over the long term, persistent deficits in walking ability necessitate an emphasis on enhancing gait efficiency and exercise tolerance through gradual increases in exercise load, posture correction, and balance training. Integrating weight management and nutritional counseling may also help reduce joint stress and optimize rehabilitation outcomes.

Taken together, these findings underscore the importance of individualized rehabilitation programs that consider each patient's physical characteristics, comorbidities, and living environment to effectively address the unique challenges faced by patients with obesity during postoperative recovery after THA.

### Limitations

This study provides valuable insights into the impact of BMI on functional recovery after THA for OA; however, several limitations should be acknowledged.

First, the relatively limited sample size warrants caution when generalizing the findings to broader populations, and specific demographic characteristics may limit external applicability. In addition, the 1-year postoperative follow-up period may not fully capture long-term functional recovery after THA.

Second, surgical techniques and rehabilitation programs were not fully standardized, reflecting real-world clinical practice but potentially introducing variability in outcomes.

Third, although both objective gait measures and subjective clinical scores were used, each assessment method has inherent limitations, and subjective evaluations may be influenced by reporting bias. Notably, while the JOA hip score includes a pain subdomain, gait-specific pain during walking (eg, visual analog scale during ambulation) was not directly assessed.

Fourth, the use of assistive devices (eg, cane or walker) during gait assessments was permitted when clinically necessary to ensure patient safety; however, the specific use of assistive devices was not systematically recorded. This may have influenced measured walking speed, particularly in the early postoperative period.

Finally, although patients with radiographically apparent adjacent joint or lumbar spine OA were excluded, subtle biomechanical influences from unmeasured joint or spinal conditions cannot be entirely ruled out.

## Conclusions

In this prospective study, we assessed the impact of BMI on functional recovery following THA for OA. Patients with obesity experienced significant delays in recovery, particularly in walking speed and stride length, during the first postoperative year. Although the JOA hip scores showed no significant differences among the BMI groups at 1 year postoperatively—indicating that subjective clinical recovery eventually equalizes—gait parameters such as walking speed and stride length did not fully normalize in the obese group, suggesting persistent objective functional limitations. These findings highlight the need for personalized rehabilitation strategies for patients with obesity to address specific challenges and optimize the long-term success of THA.

## Acknowledgments

This study was supported by the Pfizer Health Research Foundation [Grant Number: 25-Y-02].

## Conflicts of interest

The authors declare there are no conflicts of interest.

For full disclosure statements refer to https://doi.org/10.1016/j.artd.2026.101986.

## CRediT authorship contribution statement

**Musashi Ima:** Writing – review & editing, Writing – original draft, Visualization, Validation, Software, Methodology, Investigation, Formal analysis, Data curation, Conceptualization. **Tamon Kabata:** Writing – review & editing, Writing – original draft, Supervision, Resources, Project administration, Methodology, Conceptualization. **Daisuke Inoue:** Writing – review & editing, Investigation, Data curation. **Yu Yanagi:** Writing – review & editing, Investigation, Data curation. **Takahiro Iyobe:** Writing – review & editing, Validation, Investigation. **Naoya Fujimaru:** Writing – review & editing, Resources, Data curation. **Satoru Demura:** Writing – review & editing, Supervision, Formal analysis.

## Appendix A Supplementary data

Conflict of Interest Statement for Ima

Conflict of Interest Statement for Kabata

Conflict of Interest Statement for Inoue

Conflict of Interest Statement for Yanagi

Conflict of Interest Statement for Iyobe

Conflict of Interest Statement for Fujimaru

Conflict of Interest Statement for Demura
